# Active Acupoints Differ from Inactive Acupoints in Modulating Key Plasmatic Metabolites of Hypertension: A Targeted Metabolomics Study

**DOI:** 10.1038/s41598-018-36199-1

**Published:** 2018-12-13

**Authors:** Mingxiao Yang, Zheng Yu, Xiaomin Chen, Zhenyu Guo, Shufang Deng, Lin Chen, Qiaofeng Wu, Fanrong Liang

**Affiliations:** 1 College of Acupuncture and Tuina, Chengdu University of Traditional Chinese Medicine, 37 Shierqiao Road, Jinniu Street, Chengdu, 610075 Sichuan China; 2 School of Chinese Medicine, The University of Hong Kong, 10 Sassoon Road, Pokfulam, Hong Kong SAR, China; 3 Department of Chinese Medicine, The University of Hong Kong-Shenzhen Hospital, 1 Haiyuan Road, Futian District, Shenzhen, 518053 Guangdong China; 4 Metabolomics, Scientific Technology Department, BGI, Beishan Industrial Zone, Yantian District, Shenzhen, 518083 Guangdong China

## Abstract

The effect of active acupoints versus inactive acupoints in treating hypertension is not well documented. Metabolic phenotypes, depicted by metabolomics analysis, reflect the influence of external exposures, nutrition, and lifestyle on the integrated system of the human body. Therefore, we utilized high-performance liquid chromatography tandem mass spectrometry to compare the targeted metabolic phenotype changes induced by two different acupoint treatments. The clinical outcomes show that active acupoint treatment significantly lowers 24-hour systolic blood pressure but not diastolic blood pressure, as compared with inactive acupoint treatment. Furthermore, distinctive changes are observed between the metabolomics data of the two groups. Multivariate analysis shows that only in the active acupoint treatment group can the follow-up plasma be clearly separated from the baseline plasma. Moreover, the follow-up plasma of these two groups can be clearly separated, indicating two different post-treatment metabolic phenotypes. Three metabolites, sucrose, cellobiose, and hypoxanthine, are shown to be the most important features of active acupoint treatment. This study demonstrates that metabolomic analysis is a potential tool that can be used to efficiently differentiate the effect of active acupoints from inactive acupoints in treating hypertension. Possible mechanisms are the alternation of hypothalamic microinflammation and the restoration of host-gut microbiota interactions induced by acupuncture.

## Introduction

The history of acupuncture for treating cardiovascular diseases (CVD) is profound. Today, acupuncture is still one of the most popular alternative therapies for hypertension^1^. A randomized controlled trial demonstrated that acupuncture as an add-on treatment can significantly reduce 24-hour ambulatory blood pressure for essential hypertension (EH) patients^2^. Other studies have indicated that acupuncture might be particularly effective to lower blood pressure in prehypertension and stage I hypertension^3^. In addition, acupuncture has been shown to be helpful for patients with EH by improving the circadian blood pressure rhythm, which is a main indicator of EH-associated target organ damage^4^. Systematic reviews have found that acupuncture is associated with a low adverse event rate^5,6^. Basic studies show that acupuncture may reduce sympathetic outflow and reflex elevations in blood pressure by regulating neurotransmitters in the rostral ventrolateral medulla (rVLM), arcuate, and ventrolateral periaqueductal grey (vlPAG), thereby lowering blood pressure^7^. Enhanced NO/NOS activity also plays an important role in the therapeutic effect of acupuncture for hypertension^8^. However, acupoint selection is still controversial, since it is still debatable as to whether some acupoints are more specific to certain diseases (termed ‘active acupoints’) than others (‘inactive acupoints’). This is especially true for the treatment of hypertension treatment. For example, two studies compared different acupoint selection protocols and yielded opposite results^2,9^, confusing researchers and clinicians. Studies by Longhurst *et al*. addressed the difference in efficacy between active and inactive points, highlighting that the acupoint and meridian may lie within the line of nerve innervations. They went on to explain that stimulating acupoints triggers nervous reflexes, transferring the acupuncture signals to the central nervous system, yielding cardiovascular regulation^10–12^. However, existing relevant studies are insufficient to form any solid conclusions.

**Figure 1:**
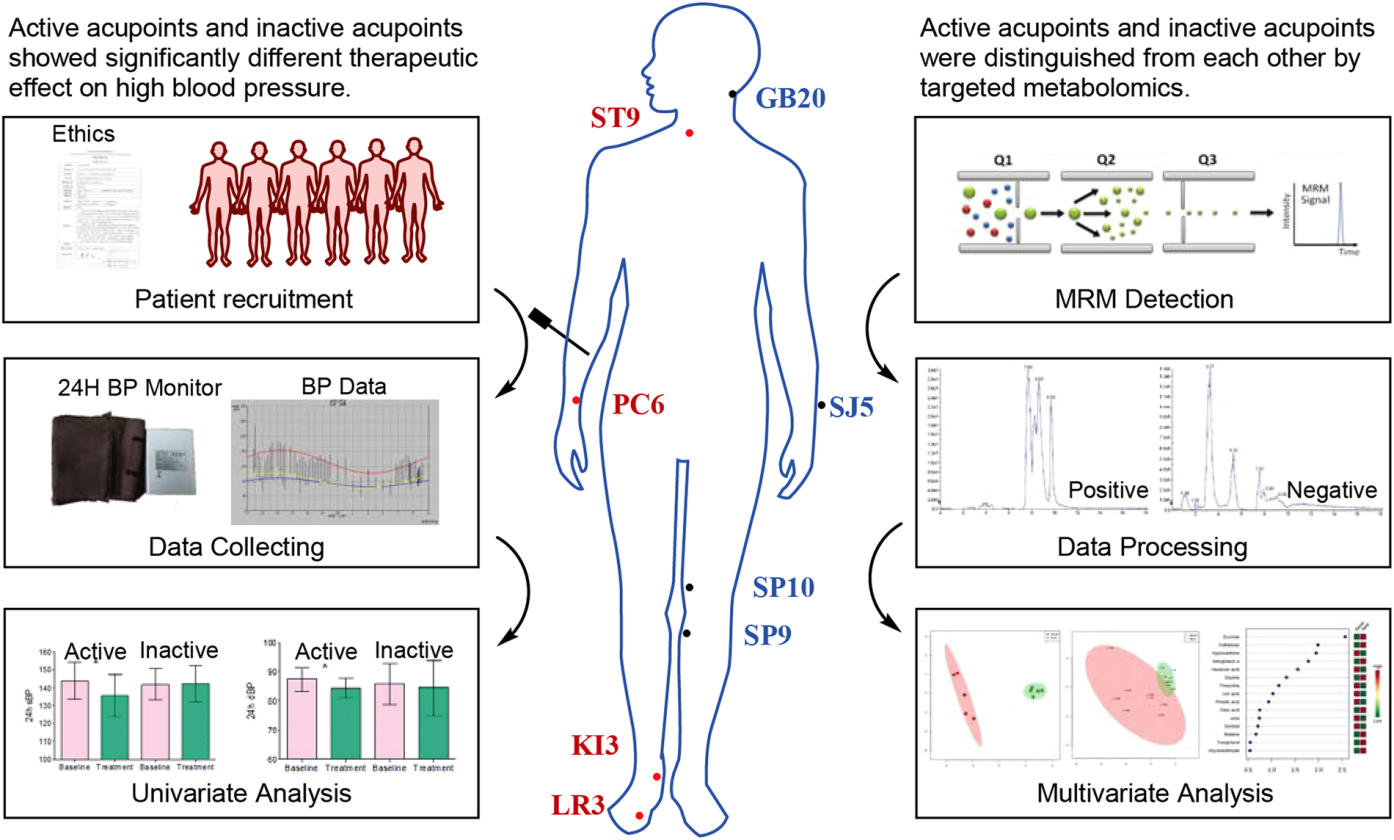
Graphic summary of the study. This study primarily assessed the blood pressure change after different acupoint treatments. A high-performance liquid chromatography tandem mass spectrometry platform was used to analyse the key plasma metabolites of hypertensive patients. The PCA and PLS-DA models were used to identify the most important features leading to the separation of metabolic phenotypes. This study shows that metabolomics methods are a potential tool to differentiate the effect of active acupoints versus inactive acupoints.

Metabolomics, which has been developed to identify small molecular metabolites within human samples by using high-throughput analytical tools, is frequently employed to characterize metabolic features and explore new diseases mechanisms^13^. In systems biology^14^, metabolomics may enable a comprehensive elucidation of the biological change within a living organism caused by environmental exposures^15^, such as nutrition or lifestyle. As such, metabolomics is used to systematically characterize the change in a metabolic profile and selecting metabolites is considered a critical tool in understanding the possible mechanism behind blood pressure regulation. Recently, metabolic dysfunction in patients with hypertension has been increasingly emphasized^16–20^. Studies demonstrate that by using metabolomic analysis, new disease biomarkers and novel metabolic pathways associated with the pathogenesis of hypertension can be identified^21,22^. Because acupuncture is a holistic therapy that exerts comprehensive therapeutic effects, its influence may involve a dynamic, network-wide alternation of multiple target genes, proteins, or metabolites^23–28^. It is therefore of great significance to elucidate the mechanism of acupuncture effects with systematic biological approaches. As such, we applied metabolomics to separate the effects generated by active acupoint treatment versus inactive acupoint treatment. The present pilot study evaluates the clinical effect of two acupoint groups and quantifies 47 CVD-related metabolites in patients’ plasma. The active acupoints were selected based on expert opinions and the inactive acupoints were used for the control. The results show different clinical effects and different metabolic regulative effects between active acupoints and inactive acupoints in lowering blood pressure (see Fig. 1**)**.

## Results

### Baseline information

**Figure 2:**
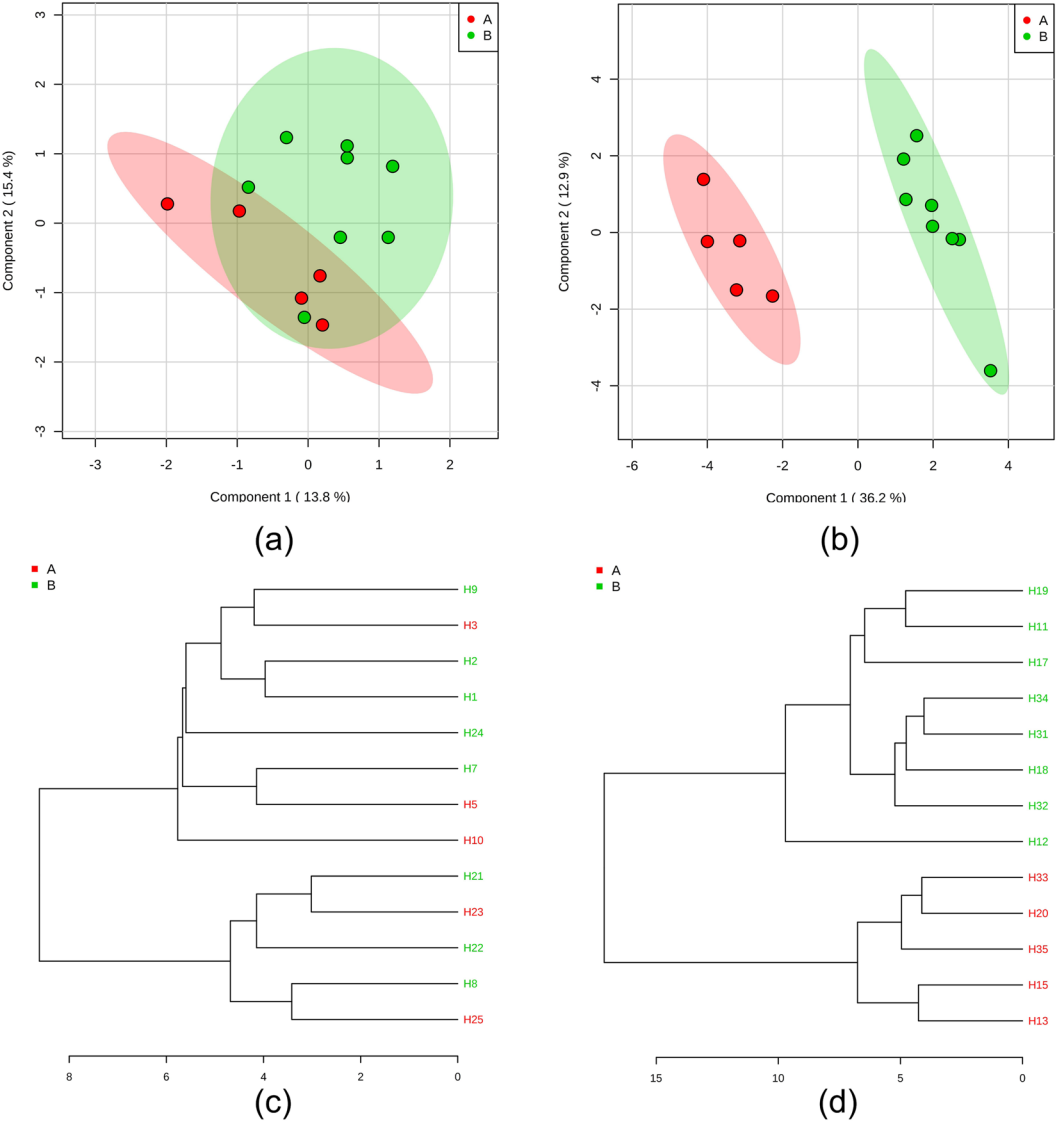
Separation of the blood sample between the two groups in the baseline and follow-up using the metabolomic data. (**a**) This PLS-DA score plot shows that in the baseline, the plasma of the two groups cannot be separated using the target metabolite information; (**b**) in the follow-up, the plasma of the two groups can clearly be separated using the target metabolite information; (**c**) in the baseline, the dendrogram cannot be used to visualize the clustering of the two groups using PLS-DA analysis; (**d**) however, after different treatments, the dendrogram shows obvious clustering of the two groups as analysed by PLS-DA.

Between 1 October, 2012, and 1 April, 2015, 103 patients were recruited from the clinical centre of the 3rd Teaching Hospital of Chengdu University of Traditional Chinese Medicine. Of the 103, only 17 eligible participants were included in the study. One patient in the active acupoints treatment group (ATG) withdrew due to immigration reasons and three patients in the inactive acupoints treatment group (ITG) discontinued their treatment—two as a result of unsatisfactory blood pressure control and one from an unexpected hip bone fracture. 13 eligible patients completed the six-week treatment. Baseline comparison showed that the two groups were well-balanced in all clinical characteristics (*P* > 0.05) (Table 1). No adverse events were reported during the trial period. Metabolomic analysis shows that at the baseline between the ATG and ITG, there is no significant difference in the target metabolites. Moreover, pattern recognition shows that the two groups cannot be separated using baseline metabolite data (Fig. 2a,c).

**Table 1:** Demographic information and blood pressure indices.

Group	ATG	ITG	*P value*
Gender (Male/Female)	3/2	5/3	0.928
Age	60.80 ± 3.40	58.88 ± 3.07	0.692
BMI	26.65 ± 1.73	25.17 ± 0.76	0.566
WHR	0.94 ± 0.02	0.89 ± 0.02	0.178
SBP	144.0 ± 10.30	142.0 ± 8.57	0.711
DBP	87.40 ± 4.10	85.88 ± 7.14	0.675
DSBP	146.80 ± 3.88	145.50 ± 2.82	0.787
DDBP	88.40 ± 1.21	88.00 ± 2.24	0.897
NSBP	135.0 ± 8.59	130.60 ± 4.08	0.614
NDBP	80.20 ± 2.11	80.25 ± 3.02	0.991
Pulse	73.00 ± 3.54	78.67 ± 1.87	0.171
Arterial BP	106.20 ± 1.50	104.70 ± 0.76	0.360
SBP Variation	12.02 ± 1.25	14.23 ± 0.81	0.159
DBP Variation	13.82 ± 2.01	19.87 ± 1.83	0.053
SBP Nocturnal Dipping	8.30 ± 3.78	9.55 ± 1.05	0.735
DBP Nocturnal Dipping	10.90 ± 1.98	9.26 ± 2.48	0.628

*ATG: active acupoint group; ITG: inactive acupoint group; BMI: body mass index; WHR: waist-height ratio; SBP/DBP: systolic/diastolic blood pressure; DSBP/DDBP: daytime systolic/diastolic blood pressure; NSBP/NDBP: night time systolic/diastolic blood pressure.

### ATG significantly reduces systolic blood pressure as compared with the ITG

After the six-week acupuncture treatment, changes in the EH patients’ blood pressure in the two experimental groups varied (Table 2). Both systolic blood pressure and diastolic blood pressure were significantly reduced after treatment for patients in the ATG (*P* < 0.05). The average arterial blood pressure was significant reduced in the ATG (*P* < 0.05). While in the ITG, which delivered inactive acupoint treatment, neither systolic nor diastolic blood pressure was significantly changed (*P* > 0.05). Only the pulse rate was significantly reduced in the ITG (*P* > 0.05). In addition, when looking at the follow-up blood pressures, active acupoints treatment can be seen to significantly lower systolic blood pressure and DBP variation, but not diastolic blood pressure, as compared with inactive acupoint acupuncture (*P* < 0.05).

**Table 2:** Blood pressure changes after six-week acupuncture treatment.

Group	Parameter	Baseline	Follow-up	MD (95% CI)	*P value*
ATG	SBP	144.0 ± 10.30	135.80 ± 11.76^Δ^	8.20(1.05, 15.35)	0.034*
	DBP	87.40 ± 4.10	84.40 ± 3.36	3.00(0.22, 5.78)	0.040*
	DSBP	146.80 ± 3.88	143.80 ± 6.14	3.00(−6.08, 12.08)	0.411
	DDBP	88.40 ± 1.21	86.60 ± 4.72	1.80(−2.96, 6.56)	0.353
	NSBP	135.0 ± 8.59	130.50 ± 18.61	4.60(−0.854, 10.05)	0.079
	NDBP	80.20 ± 2.11	76.80 ± 3.11	3.40(−5.36, 12.16)	0.342
	Pulse	73.00 ± 3.54	77.20 ± 6.76	−4.20(−8.54, 0.14)	0.055
	Arterial BP	106.20 ± 1.50	103.0 ± 2.45	3.20(1.58, 4.82)	0.005*
	SBP Variation	12.02 ± 1.25	14.58 ± 3.12	−2.56(−8.44, 3.32)	0.293
	DBP Variation	13.82 ± 2.01	15.83 ± 2.58	−2.00(−9.29, 5.26)	0.485
	SBP Nocturnal Dipping	8.30 ± 3.78	6.18 ± 9.95	2.11(−7.08, 11.3)	0.558
	DBP Nocturnal Dipping	10.90 ± 1.98	11.10 ± 6.07	−0.20(−10.98, 10.58)	0.961
ITG	SBP	142.0 ± 8.57	142.30 ± 10.21	−0.25(−6.92, 6.42)	0.932
	DBP	85.88 ± 7.14	84.63 ± 9.41	1.25(−4.24, 6.74)	0.607
	DSBP	145.50 ± 2.82	144.60 ± 11.16	0.88(−6.49, 8.24)	0.787
	DDBP	88.00 ± 2.24	86.13 ± 9.43	1.88(−3.73, 7.48)	0.455
	NSBP	130.60 ± 4.08	135.30 ± 8.92	−4.63(−11.10, 1.85)	0.135
	NDBP	80.25 ± 3.02	79.88 ± 10.84	0.38(−7.55, 7.30)	0.902
	Pulse	78.67 ± 1.87	78.67 ± 4.59	6.17(0.84, 11.49)	0.030*
	Arterial BP	104.70 ± 0.76	104.20 ± 7.36	0.50(−6.33, 7.33)	0.858
	SBP Variation	14.23 ± 0.81	14.64 ± 4.03	−0.40(−4.89, 4.09)	0.827
	DBP Variation	19.87 ± 1.83	19.66 ± 2.24^∆^	0.21(−6.44, 6.86)	0.938
	SBP Nocturnal Dipping	9.55 ± 1.05	6.44 ± 4.91	3.11(−1.62, 7.84)	0.152
	DBP Nocturnal Dipping	9.26 ± 2.48	6.54 ± 7.12	2.71(−7.17, 12.60)	0.512

* indicates P < 0.05; ^∆^P < 0.05 which is yielded in the comparison between Group A and Group B after treatment.

### Target metabolomics separated the follow-up plasma from the baseline plasma in the ATG

**Figure 3:**
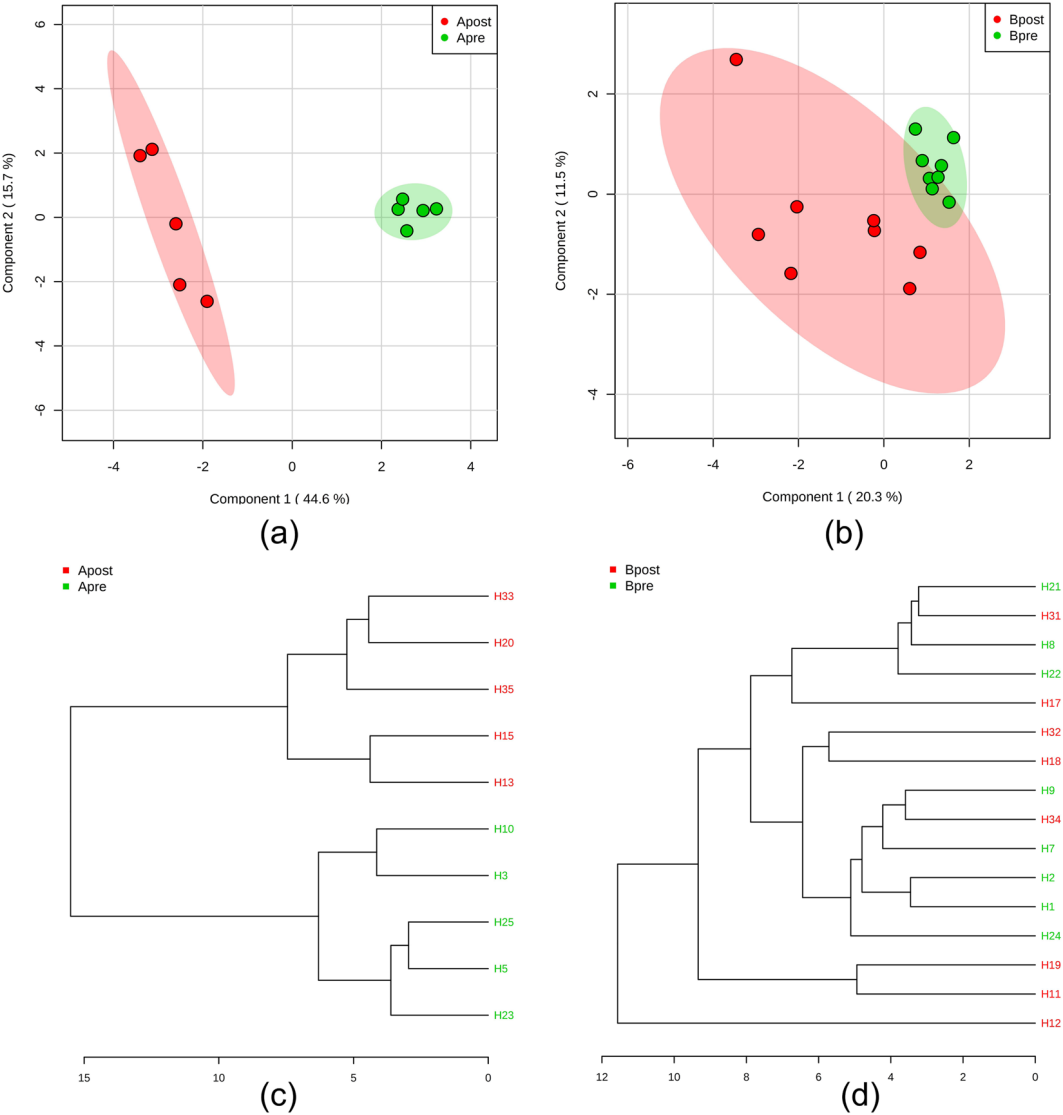
Separation of the blood sample between the baseline and follow-up in the two different groups. (**a**) This PLS-DA score plot shows that in the ATG, the plasma of two time points can be easily and clearly separated using the target metabolite information; (**b**) in the ITG, the plasma of two time points cannot be separated from each using the target metabolite information; (**c**) in the ATG, the dendrogram shows the clustering of the two groups using PLS-DA analysis; (**d**) while in the ITG, the dendrogram does not show significant clustering of the two groups.

According to the results of the fold change (threshold >=2) analysis and the t-tests (threshold =<0.05), eight key plasmatic metabolites in the ATG, including sucrose, cellobiose, glycine, hypoxanthine, hexanoic acid, ketoglutaric acid, threonine, and uric acid, were significantly altered, and thus were selected as important features. However, in the ITG only one metabolite, ketoglutaric acid, showed significant change. The acupuncture-induced key plasmatic metabolite changes are listed in Table 3. PCA and PLS-DA clearly separated the baseline EH plasma from the follow-up EH plasma in the ATG, as shown in the PLS-DA score plot (Fig. 3a) and the dendrogram (Fig. 3c). However, in the ITG, follow-up plasma cannot be distinguished from baseline plasma (Fig. 3b,d). Five key metabolites, including sucrose, cellobiose, hypoxanthine, ketoglutaric acid, and hexanoic acid, were selected as markers for the ATG by using a VIP threshold of 1.5. These metabolites, except for ketoglutaric acid, were recognized as signature metabolites in active acupoint treatment. As for the ITG, ketoglutaric acid and betaine were selected by employing the same threshold.

**Table 3:** Acupuncture-induced changes to key metabolites selected by t-tests and FC analysis.

Group	Metabolites	Baseline	Follow-up	log2(FC)	*P* value	−log10(p)
ATG	Sucrose	19.80 ± 6.49	1.88 ± 1.24	−3.5686	4.56E-05	4.3408
	Cellobiose	429.2 ± 227.6	87.28 ± 36.90	−2.5744	0.000964	3.0158
	Glycine	1482 ± 259.1	825.6 ± 261.6	−1.0927	0.001052	2.9781
	Hypoxanthine	3.73 ± 1.32	25.14 ± 18.92	2.5342	0.00134	2.8728
	Hexanoic acid	53.83 ± 20.40	184.4 ± 65.22	1.6189	0.004884	2.3112
	ketoglutaric acid	5.04 ± 3.43	1.34 ± 0.93	−2.1528	0.009107	2.0406
	Threonine	3.57 ± 1.18	8.36 ± 3.92	1.0422	0.017975	1.7453
	Uric acid	17.61 ± 3.54	45.03 ± 26.95	1.2089	0.083333	1.0792
ITG	ketoglutaric acid	5.16 ± 1.72	21.95 ± 16.44	1.8934	0.006078	2.2162

### Post-treatment metabolic phenotypes of patients in the ATG differed from the ITG

PCA and PLS-DA analysis demonstrates that the follow-up plasma of EH patients receiving active acupoint acupuncture can be separated from patients receiving inactive acupoint acupuncture, as shown in PLS-DA score plot (Fig. 2b). It demonstrates the different effect between active acupoints and inactive acupoints on key metabolites. The clustering result shown in the dendrogram also suggests a clear separation between the ATG and ITG (Fig. 2d). Using a VIP threshold of 1.5, ketoglutaric acid, sucrose, cellobiose, and hypoxanthine were selected as important features in differentiating the follow-up plasma of the ATG from the ITG. Results indicate that sucrose, cellobiose, and hypoxanthine can be signature metabolites highlighting the effect of active acupoints in the treatment of EH. Ketoglutaric acid, uric acid, and pimelic acid are candidate key metabolites for active acupoints.

## Discussion

In this study, we employ a combing omics and comparative effectiveness research model to address the controversy between active and inactive acupoints—an innovative, evidence-based clinical research decision-making model for Chinese medicine^29^. This model is especially advantageous for research into complementary and alternative medicine, because not only does it efficaciously produce clinical evidence for decision making based on patient-centred outcomes, but it also sheds light on possible molecular mechanisms. The results show that active acupoints, selected on the basis of traditional theories, is more effective than disease-unrelated inactive acupoints in the lowering of blood pressure. Based on the metabolomic analysis, metabolic modulation may also play a significant role.

**Figure 4:**
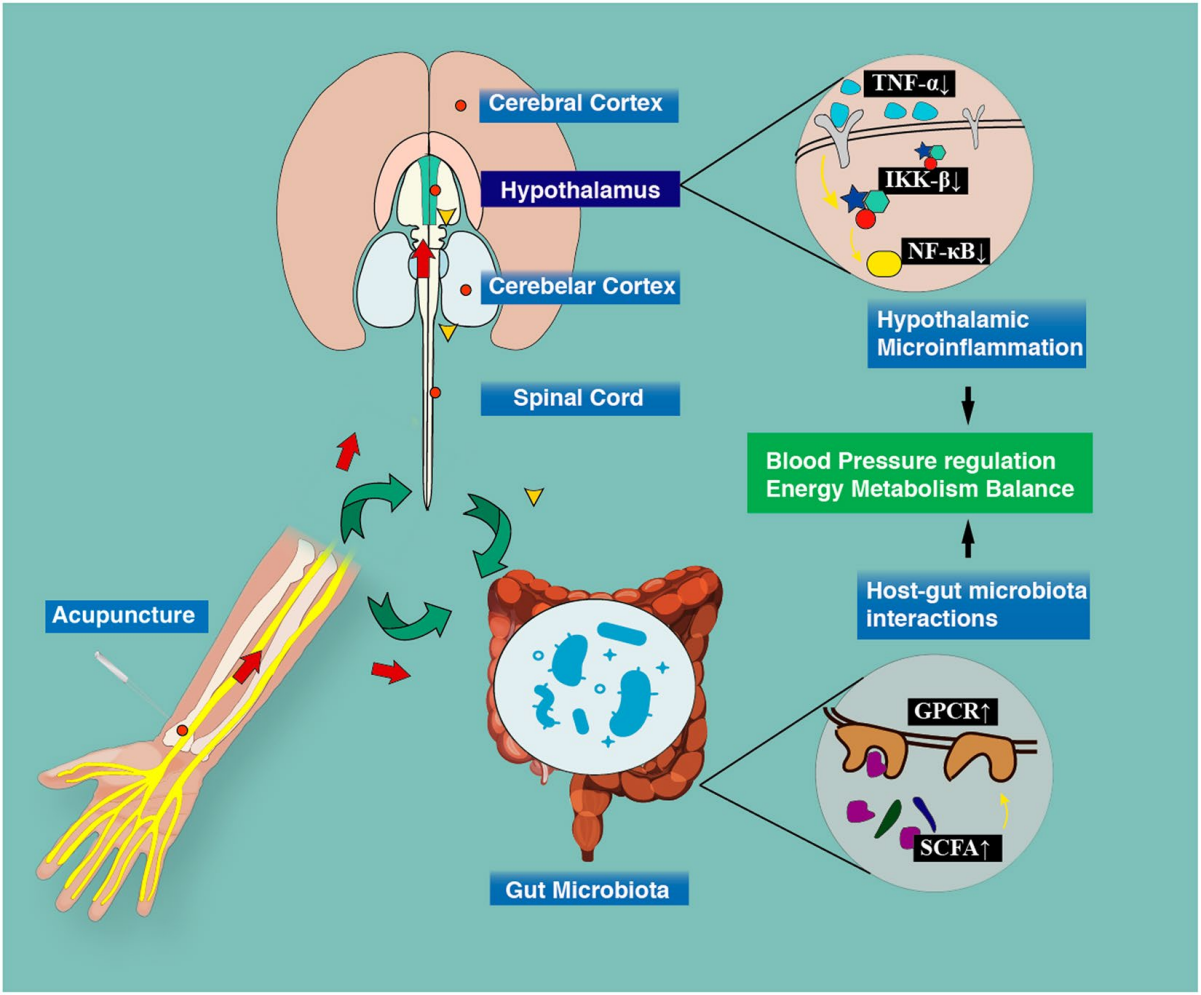
Potential mechanism of acupuncture improving blood pressure and metabolic function. Acupuncture signals can be transferred from peripheral nerves to the central nervous system. This study indicates that the alternation of hypothalamic microinflammation and the restoration of host-gut microbiota interactions induced by acupuncture could be a possible mechanism for acupuncture to lower blood pressure and restore metabolic abnormality.

One possible mechanism indicates that it is highly likely that acupuncture simultaneously improves blood pressure and lipid/glucose metabolism by inhibiting hypothalamus microinflammation. Many studies have indicated that this physiological process is the central mechanism of metabolic dysfunctions in hypertension^30^. Active acupuncture stimulation might trigger afferent nerves innervated beneath active acupoints to transfer mechanical and biochemical signals to the brain areas responsible for blood pressure regulation, including the hypothalamus, rVLM, and vlPAG^31^. Studies have also confirmed that acupuncture can inhibit reflex hypertension through the opioid-mediated inhibition of glutamate in rVLM^32^. Therefore, glutamatergic suppression may further reverse the hypertensive effect of central TNF-α, which simultaneously recovers blood pressure and energy balance via inhibiting NF-κB and IKK β activation^33,34^ (Fig. 4).

Moreover, sucrose, cellobiose, and hypoxanthine were considered signature metabolites of active acupoints for hypertension. Hypoxanthine is the precursor of uric acid—the end product of purine degradation in humans^35^. High plasma uric acid is a precipitating factor in gout and renal calculi, as well as a strong risk factor for metabolic syndrome and cardiovascular disease^36^. Studies demonstrate that serum uric acid can be a sensitive biomarker for blood pressure elevation^37^. It can also be used to predict the onset of out-of-office hypertension, as well as long-term cardiovascular and all-cause mortality^38,39^. Our study shows that active acupoints treatment increases the level of hypoxanthine, indicating a suppression of purine degradation and uric acid production. Sucrose is consumed by humans to provide sufficient energy and is broken down into the constituent monosaccharides of glucose and fructose by sucrase or isomaltase glycoside hydrolases in the membrane of the microvilli lining in the duodenum^40,41^. The use of sucrose as a sweetening agent in food and beverages is becoming increasingly frequent. However, evidence suggests that sucrose poses a threat to human health by increasing the risk of cardiovascular diseases and metabolic syndrome^42,43^. Recent studies suggest that six months consumption of sucrose-sweetened soft drinks (SSSDs) increases the risk of metabolic complications in overweight and obese subjects^44^. Another study suggests that a six-month daily intake of SSSDs increases ectopic fat accumulation and lipids, as compared with milk, diet cola, and water^45^. In our study, the plasma sucrose level was significantly reduced, suggesting that acupuncture may inhibit the duodenal absorption of sucrose, or accelerate its anabolism to improve metabolic abnormality.

Furthermore, gut-microbiota may participate in the blood pressure regulation mechanisms of acupuncture. This study finds that active acupoint treatment also reduces the level of cellobiose, which is fermented in the large intestine of humans^46^. Cellobiose is a prebiotic ingredient in fermented products involving *bifidobacterial*^47^. Gut microbiota, as a major part of the commensal microbiome in humans, plays a significant role in maintaining intestinal hemostasis. It may also interact with human metabolism via enterohepatic circulation, as well as many other immunoendocrine mechanisms, and thus contribute to the maintenance of hemostasis. Various studies demonstrate that disruption of the gut microbiota (dysbiosis) is either causally or consequently associated with a variety of diseases, including Crohn’s disease, colon cancer, diabetes, metabolic syndrome, cardiovascular disease, stress and anxiety, food allergies, asthma, autism, hepatic encephalopathy, and eczema^48^. Mounting evidence supports the role of gut microbiota in the development and maintenance of hypertension^49^. Research indicates that short-chain fatty acids and other small molecular metabolites produced by gut microbiota bind to metabolite-sensing G-protein coupled receptors (GPCRs), such as GPR41, GPR43, and GPCR olfactory receptor 51E2 (humans)/Olfr78 (mice), to regulate blood pressure via controlling gut hemostasis, host metabolism, and immune response. Our previous study showed that in healthy males, acupuncture significantly increased concentrations of urinary hippuric acid^50^, which is a co-metabolite in humans and gut microbiota, and a possible biomarker of hypertension and obesity in humans^48,51,52^. Future studies are needed to explore this mechanism.

It should be noted that the current comparison is only validated by this pilot study. Its power may be limited by the small sample size, even though the antihypertensive effect observed (systolic BP reduction: 8.2 mmHg) is equivalent to that of a relatively large trial (10.8 mmHg)^53^. It remains unclear if an increase of sample size will dilute this clinical efficacy. Moreover, there is a further need for the assessment of acupuncture’s long-term effect on hypertension. Mounting evidence shows that acupuncture has persisting therapeutic effects on chronic pain^54,55^. However, it is still largely unknown whether this effect remains when used for hypertension. Current research measures the immediate BP reduction following acupuncture treatment. For example, Flachskampf *et al*. reveals that the hypotensive effect of acupuncture could be time-dependent^2^. It also shows that the clinical efficacy detected at the completion of the treatment did not last for more than 3–6 months after treatment. These questions will be addressed in our future studies.

In conclusion, metabolomic analysis is a potential tool that can be used to effectively differentiate the effect of active acupoints from inactive acupoints in treating hypertension. Possible mechanisms are the alternation of hypothalamic microinflammation and the restoration of host-gut microbiota interactions induced by acupuncture.

## Methods

### Ethics, consent and permissions

This study has been ethically reviewed by the Sichuan Regional Ethical Review committee of the Teaching Hospital of Chengdu University of Traditional Chinese Medicine (NO. 2012KL-003). All trial procedures were designed and conducted in accordance with the Declaration of Helsinki. Patients’ right and beneficence were placed at the utmost place in this study. This study was registered in the NIH ClinicalTrial.gov Registry (ClinicalTrials.gov Identifier: NCT03492892, 09/04/2018). Each eligible participant was informed about all the procedures, benefits as well as potential risks that they may encounter in this trial, and they are free to withdraw the study at any time without any specific reason. Before any trial process, the included participants were required to submit a written inform consent signed by oneself or their next of kin.

### Participants

Patients of essential hypertension were included and clear diagnosis of EH was made according to the seventh report of the joint national committee on prevention, detection, evaluation, and treatment of high blood pressure, the JNC 7 report^56^. Study participants were recruited from the third teaching hospital of Chengdu University of Traditional Chinese Medicine and its surrounding communities. Patients meet all inclusion criteria are included in this study, any violation of the exclusion criteria made participants excluded. Inclusion criteria: (1) aged between 45 and 75 years; (2) were diagnosed as stage I hypertension in the first visit, or used to be diagnosed as stage I hypertension in recent 1 year, but without any medication history; (3) without neurological, other cardiovascular, hepatic and renal disease, and other visceral diseases; (4) the basal metabolism rate of a patient, which was measured by the retrospective dietary questionnaire at baseline, should be approximately 1:1.4; (5) didn’t administer any drugs or herbs in at 15 days before the start of the study; (6) didn’t participate in any study other than this; (7) agreed to cooperate with researchers in all research procedures after they were introduced this study; and (8) provided with written informed consent. Exclusion criteria: (1) age ≤45 or age ≥75; (2) with hypertension which was secondary to other diseases, such as renal vascular disease, Cushing’s syndrome, hyperadrenocorticism and drug-induced hypertension; (3) had complicated cardiovascular, digestive, respiratory, urinary, blood, nervous, endocrine system and other severe primary diseases and failed to effectively control in clinic; (4) accompanied by epilepsy, sleep apnea hypopnea syndrome, etc.; (5) with psychiatric symptoms such as severe depression or anxiety (SAS ≥ 70, or SDS ≥ 72); (6) pregnant or lactating woman, or woman of reproductive age who was intended to conceive in recent 1 year; (7) with abnormality in laboratory test of blood biochemistry, or with contagious risks, such as HIV virus carrier, or patient with positive HBV superficial antigen; (8) with malignant tumor or other severe consuming diseases, or patient with infections or bleeding disorders; (9) alcoholics or drug abusers, or vegetarians; (10) used to suffer from acute diseases in recent 2 weeks, such as high fever, or gastritis; (11) used to administer any drug that may potentially impaired renal or hepatic function; (12) with cardiovascular disease that had been treated with acupuncture within recent three months; or (13) undergoing other clinical trials.

### Study schedules

This study included the following procedures: patient recruitment, baseline information collection and blood sampling, randomization and allocation (1:1), treatment and outcome evaluation. The 24-hour ambulatory blood pressure was measured at baseline period and follow-up phase by an oscillometric device (A&D Co.Ltd., Japan TM-2430) within 24 hours before the commencement, and after the completion of acupuncture treatment, respectively. Venous blood was collected the day before acupuncture treatment and the second day of the final treatment at 8:00–9:00 a.m. with all the subjects having fasted for 12 hours. Fasting blood samples were collected into vacutainer tubes with heparin sodium and immediately cool to 4 °C. Then they were centrifuged within 2 h at 1500 rpm for 15 min. Plasma was separated, transferred to new vials, and immediately stored frozen (−80 °C) until preparation. The randomization procedure was done by an independent researcher with a random number table. Patients were not aware of which type of treatment they received. The primary outcome is the 24-hour ambulatory blood pressure change after treatment.

### Interventions

After inclusion, patients were allocated to either ATG or ITG. The principle of active and inactive acupoints selection is based on a recent clinical trial^57^. In ATG, four acupoints, taichong (LR3), renying (ST9), taixi (KI3), and neiguan (PC6) were selected. While, for ITG, fengchi (GB20), waiguan (SJ5), yinlingquan (SP9), and xuehai (SP10) were stimulated. All acupoints were gently superficially inserted with filiform needles bilaterally. Patients have been induced to get Deqi sensation after slightly rotating, lifting and thrusting the needle. Then the needle tail was connected with Han’s acupoints nerve stimulator, HANS-200, (Nanjing, China) to maintain the Deqi sensation. The whole needle retention time is 30 min. In each group, patients received acupuncture treatment 3 times a week for a total of 6 weeks. All practitioners have at least 5 years’ clinical experience of acupuncture.

### Target metabolomics analysis

47 target metabolites (L-Tyrosine, L-Phenylalanine, L-Threonine, L-(+)-Lactic acid, L-Valine, L-Leucine, L-Proline, Betaine, Palmitic acid, Stearic acid, Glycine, (±)-a-Tocopherol, β-Sitoseterol, L-Tryptophan, DL-glyceraldehyde, Glycocholic acid, oleic acid, eicosanoic acid, hexanoic acid, Heptanoic acid, nonanoic acid, Galactose, Sucrose, Sorbitol, myoinositol, Fructose, Cellobiose, Urea, Isoleucine, Alanine, citric acid, Azelaic acid, Aspartic acid, 4-Hydroxybenzoic acid, Pimelic acid, L-Serine, Hypoxanthine, D-Homoserine, Uric acid, Trimethylamine oxide, Pentanedioic acid, Allantoin, Linoleic acid, Citrulline, Oxaloacetic acid, and Sorbose andα-ketoglutaric acid) were selected from previous studies^58–67^ that reported new biomarkers or important metabolites for EH and related cardiovascular diseases. LC-MS grade standard compounds were purchased from Sigma-Aldrich Company (St. Louis, MO). We then detected their content by using the Multiple Reaction Monitoring (MRM) Mass-Spectrometry (MS) measurement. The analyzes for all the experiments were performed on a QTRAP5500 mass spectrometer (AB SCIEX, Framingham, MA, USA) equipped with a Shimadzu UFLC system, which consisted of Shimadzu LC-20AD XR pumps and an SIL-HTC autosampler (Kyoto, Japan). Two microliters of each sample were injected on a Luna 5 μm, 150 mm × 4.6 mm HPLC column (Phenomenex, Torrance, CA). The mobile phases were: (A) 0.01% heptafluorobutyric acid, 0.1% formic acid in water, and (B) 0.01% heptafluorobutyric acid, 0.1% formic acid in methanol. The needle-rinse solvent was methanol. The HPLC flow rate was set at 0.8 mL/min and eluted with a gradient of 2–40% solvent B for 6 min, 40% solvent B for 4 min, 40–90% solvent B for 0.5 min, 90% solvent B for 0.5 min, 90-2% solvent B for 1 min, and followed by 2% solvent B for 3 min. The column was re-equilibrated with 2% B for 1 min prior to the next injection. In QTRAP5500, the parameters were set to positive ionization mode, with curtain gas set at 40 psi, nebulizer gas at 60 psi, IonSprayTM voltage at 5500 V, ion source temperature at 600 °C, and CAD gas at medium and to negative ionization mode, with curtain gas set at 40 psi, nebulizer gas at 25 psi, IonSprayTM voltage at −4500 V, ion source temperature at 600 °C, and CAD gas at medium. The MRM transitions for the analyzes, declustering potential (DP), entrance potential (EP), collision energy (CE), and the collision cell exit potential (CXP) are listed in Supplementary Table 1. Data processing was carried out using Analyst software (AB SCIEX, version 1.6.1). The lowest limit of quantitation (LLOQ) was determined as the lowest detected concentration with the coefficient of variation less than 10%. The instrument lower limit of detection (LLOD) was based on a signal-to-noise value greater than 3. The raw data of MRM was processed using Multiquant software 2.0.2. SignalFinder1 (AB SCIEX) was applied to calculate the corresponding peak areas of MRM signals.

### Statistical analysis

Comparisons of clinical characteristics between ATG and ITG were performed by using the unpaired t-test. While for the comparison of baseline indices with post-treatment indices, the paired t-test was applied. The significance level for comparison is *P* < 0.05. As to target metabolomics analysis, we applied PCA and PLS-DA statistics to visualize the change of key metabolites’ distribution patterns after different treatment. Preliminary data were median-normalized, log-transformed and pareto-scaled prior to being introduced to the online software MetaboAnalyst (http://www.metaboanalyst.ca/MetaboAnalyst/). PCA was applied to show the distribution pattern of samples, and to detect data outlier to exclude. PLS-DA is a supervised statistical method to which the separating information is already known. It helps to figure out important features contributing to separation. Variable Importance in Projection (VIP) scores estimate the importance of each variable in the projection used in a PLS model and is often used for variable selection. A variable with a VIP Score equivalent to or greater than 1 (one) can be considered important in given model. In this case, we applied a VIP score threshold 1.5. Important features were selected by VIP score with threshold 1.5, t-tests with threshold 0.05, and fold-change analysis with threshold 2.

## Electronic supplementary material

Supplementary dataset

## Data Availability

The datasets generated during and/or analyzed during the current study are available from the corresponding author on reasonable request.
