# Supplementary material for: Active Acupoints Differ from Inactive Acupoints in Modulating Key Plasmatic Metabolites of Hypertension: A Targeted Metabolomics Study
Source: Sci Rep. 2018 Dec 13;8:17824. doi: 10.1038/s41598-018-36199-1 (PMC6292875; doi:10.1038/s41598-018-36199-1)
Supplement: Supplementary file 1 — Supplementary dataset [file 41598_2018_36199_MOESM1_ESM.docx]

**Active Acupoints Differ from Inactive Acupoints in Modulating Key Plasmatic Metabolites of Hypertension: A Targeted Metabolomics Study**

**Mingxiao Yang, Zheng Yu, Xiaomin Chen, Zhenyu Guo, Shufang Deng, Lin Chen, Qiaofeng Wu^†^, Fanrong Liang^†^**

## Supplemental Table

## Table1. MRM transitions for the analytes, declustering potential (DP), entrance potential (EP), collision energy (CE) and the collision cell exit potential(CXP)

| **Analyte** | | **Q1** | **Q3** | **DP** | **EP** | **CE** | **CXP** |
| --- | --- | --- | --- | --- | --- | --- | --- |
| Betaine | 1 | 118.5 | 58.9 | 200 | 10 | 28.4 | 13 |
|  | 2 | 118.5 | 73.1 | 100 | 10 | 25 | 13 |
| Hexanoic acid | - | 117.7 | 42 | 200 | 10 | 70.67 | 13 |
| Oxaloacetic acid | 1 | 133.1 | 70 | 100 | 10 | 24.2 | 13 |
|  | 2 | 133.1 | 87 | 200 | 10 | 14.9 | 13 |
| D-Sorbitol | 1 | 183.1 | 69 | 100 | 10 | 19.94 | 13 |
|  | 2 | 183.1 | 129 | 100 | 10 | 11.79 | 13 |
|  | 3 | 183.1 | 147 | 100 | 10 | 10.63 | 13 |
| Pimelic acid | - | 161.3 | 69.1 | 100 | 10 | 22.02 | 13 |
| Hypoxanthine | 1 | 137 | 55 | 250 | 10 | 43 | 10 |
|  | 2 | 137 | 81.9 | 200 | 10 | 33 | 10 |
|  | 3 | 137 | 119 | 230 | 10 | 28 | 10 |
| D-Homoserine | 1 | 120.7 | 57 | 60 | 10 | 23.49 | 13 |
|  | 2 | 120.7 | 75.1 | 70 | 10 | 14.95 | 13 |
| Uric acid | 1 | 169 | 141 | 130 | 10 | 21.75 | 13 |
|  | 2 | 169 | 152.1 | 130 | 10 | 23.45 | 13 |
|  | 3 | 169 | 70.1 | 130 | 10 | 31.34 | 13 |
| Isoleucine | 1 | 132 | 86 | 50 | 10 | 14.9 | 8 |
|  | 2 | 132 | 69.1 | 50 | 10 | 23.3 | 8 |
|  | 3 | 132 | 44.1 | 50 | 10 | 32.1 | 8 |
| L-Tyrosine | 1 | 182.1 | 119 | 80 | 10 | 23.76 | 13 |
|  | 2 | 182.1 | 136.1 | 40 | 10 | 18.1 | 8 |
|  | 3 | 182.1 | 165.1 | 40 | 10 | 13.1 | 8 |
| L-Phenylalanine | 1 | 166.1 | 103.1 | 100 | 10 | 35 | 13 |
|  | 2 | 166.1 | 77.1 | 128 | 10 | 50.2 | 8 |
|  | 3 | 166.1 | 120 | 50 | 10 | 31.7 | 8 |
| L-Tryptophan | 1 | 205 | 146 | 150 | 10 | 23.65 | 13 |
|  | 2 | 205 | 118 | 45 | 10 | 34.4 | 8 |
|  | 3 | 205 | 115.1 | 45 | 10 | 49 | 8 |
| L-Leucine | 1 | 132.1 | 86.1 | 50 | 10 | 14.9 | 8 |
|  | 2 | 132.1 | 43.1 | 100 | 10 | 35.1 | 13 |
|  | 3 | 132.1 | 44.1 | 50 | 10 | 29.5 | 8 |
| L-Threonine | 1 | 120.1 | 102.1 | 80 | 10 | 15.07 | 8 |
|  | 2 | 120.1 | 56 | 30 | 10 | 24 | 8 |
|  | 3 | 120.1 | 74 | 30 | 10 | 14 | 8 |
| L-Vline | 1 | 118.1 | 57 | 100 | 10 | 12.01 | 13 |
|  | 2 | 118.1 | 72.1 | 40 | 10 | 15.8 | 8 |
|  | 3 | 118.1 | 55.1 | 40 | 10 | 29.6 | 8 |
| Glycine | 1 | 76 | 30 | 50 | 10 | 24.8 | 8 |
|  | 2 | 76 | 48 | 50 | 10 | 11.2 | 8 |
| urea | 1 | 61.4 | 44 | 100 | 10 | 24.17 | 13 |
|  | 2 | 61.4 | 45 | 100 | 10 | 26.21 | 13 |
| Citrulline | 1 | 175.5 | 114.1 | 70.92 | 10 | 21 | 13 |
|  | 2 | 176.1 | 159 | 40 | 10 | 15 | 8 |
|  | 3 | 176.1 | 70.1 | 40 | 10 | 28.8 | 8 |
| Sucrose | 1 | 341.2 | 178.9 | -100 | -10 | -16.8 | -21 |
|  | 2 | 341.2 | 89 | -100 | -10 | -28 | -21 |
|  | 3 | 341.2 | 119 | -100 | -10 | -21 | -21 |
| D-(+)-Galactose | 1 | 178.8 | 58.9 | -100 | -10 | -22 | -22 |
|  | 2 | 178.8 | 70.7 | -100 | -10 | -23 | -23 |
|  | 3 | 178.8 | 88.6 | -100 | -10 | -11 | -11 |
| myo-Inositol | 1 | 178.8 | 160.9 | -100 | -10 | -15.01 | -13 |
|  | 2 | 178.8 | 87 | -100 | -10 | -22.95 | -13 |
|  | 3 | 178.8 | 116.8 | -100 | -10 | -19.01 | -13 |
| D-(−)-Fructose | 1 | 179.1 | 58.8 | -80 | -10 | -17 | -13 |
|  | 2 | 179.1 | 71 | -80 | -10 | -16 | -13 |
|  | 3 | 179.1 | 89 | -80 | -10 | -11 | -13 |
| D-(+)-Cellobiose | 1 | 341.3 | 161 | -100 | -10 | -9.59 | -21 |
|  | 2 | 341.3 | 179 | -100 | -10 | -10.41 | -21 |
|  | 3 | 341.3 | 101 | -100 | -10 | -19.88 | -21 |
| α-ketoglutaric acid | 1 | 145.3 | 57.1 | -10 | -14.95 | -14 | -14 |
|  | 2 | 145.3 | 73 | -10 | -19.34 | -14 | -14 |
|  | 3 | 145.3 | 101.2 | -10 | -18 | -14 | -14 |
